# The tumour microenvironment of pilocytic astrocytoma evolves over time via enrichment for microglia

**DOI:** 10.1186/s40478-024-01922-9

**Published:** 2025-02-13

**Authors:** Thomas J. Stone, Jessica C. Pickles, Olumide Ogunbiyi, Shireena A. Yasin, Catherine A. Taylor, Saira W. Ahmed, Jane Chalker, Carryl Dryden, Iwona Slodkowska, Emily Pang, Mark Kristiansen, Rachel Williams, Helena Tutill, Charlotte A. Williams, Gaganjit K. Madhan, Leysa Forrest, Tony Brooks, Mike Hubank, Debbie Hughes, Paula Proszek, Grzegorz Pietka, Erin Peat, Darren Hargrave, Thomas S. Jacques

**Affiliations:** 1 Developmental Biology and Cancer Research and Teaching Department, UCL Great Ormond Street Institute of Child Health, 30 Guilford Street, London, WC1N 1EH UK; 2 UCL Genomics, Zayed Centre for Research into Rare Disease in Children, 20 Guilford Street, London, WC1N 1DZ UK; 3 Department of Histopathology, Great Ormond Street Hospital for Children NHS Foundation Trust, Great Ormond Street, London, WC1N 3JH UK; 4 Specialist Integrated Haematology and Malignancy Diagnostic Service, Great Ormond Street Hospital for Children NHS Foundation Trust, Great Ormond Street, London, WC1N 3JH UK; 5 Department of Haematology and Oncology, Great Ormond Street Hospital for Children NHS Foundation Trust, Great Ormond Street, London, WC1N 3JH UK; 6 Clinical Genomics, Centre for Molecular Pathology, Royal Marsden Hospital, London, SM2 5NG UK

**Keywords:** Low-grade glioma, Pilocytic astrocytoma, Microglia, Immune microenvironment

## Abstract

Pilocytic astrocytoma (PA) is the commonest low-grade tumour affecting children and is frequently experienced as a chronic disease associated with extended treatment, periods of regrowth, and long-term disability. This contrasts with the view of PA as a benign tumour with positive clinical outcomes and raises the fundamental question of biologically driven change over time within these tumours, which will impact diagnosis, stratification, and management. To investigate the molecular, cellular, and pathological stability of PA we performed RNA sequencing, methylation array profiling, immunohistochemistry, and targeted panel DNA sequencing on a cohort of 15 PA patients with matched primary/longitudinal samples at a mean sampling interval of 2.7 years. Through pairwise analysis of primary versus longitudinal tumour samples we identified changes to immune-related pathways within the expression and methylation profiles of longitudinal PA. Further interrogation of these changes revealed an enrichment over time for microglial cell populations, which was validated by immunohistochemistry against common monocyte/microglial markers. Moreover, immunohistochemical characterisation revealed concurrent increases in the expression of M2-like and anti-inflammatory markers. Microglial enrichments were consistent across the cohort and were not adequately explained by a range of potential confounders, including receipt of adjuvant therapy. Taken together, these data challenge the idea of pilocytic astrocytoma as a static entity and indicate that they consistently accumulate microglia over time, potentially co-opting the immune microenvironment towards an anti-inflammatory phenotype that may affect the natural course and treatment response of the tumours.

**Supplementary Information:**

The online version contains supplementary material available at 10.1186/s40478-024-01922-9.

## Introduction

Pilocytic astrocytomas (PA) are the commonest low-grade CNS tumours affecting children and alongside other paediatric low-grade glioma they are considered a single pathway disease driven by alterations in the MAPK/ERK pathway, most frequently a *KIAA1549::BRAF* fusion [29, 30]. Due to their classification as a low-grade tumour, a common view of PA is that they are benign tumours with positive outcomes. However, this outlook is misleading if one considers patient experience and quality of life throughout the disease course. Patients frequently suffer long-term physical and cognitive disabilities, with one large cohort reporting that 59% of paediatric PA patients required special education services 3 years after diagnosis [1, 36, 49]. Notably, risk factors for disability include younger age at diagnosis and relapse [1]. This is particularly relevant as a proportion of patients will experience PA as a chronic disease with periods of regrowth [2, 15, 33]. In one large retrospective cohort, after the exclusion of pilomyxoid astrocytoma, 25% of patients with PA experienced at least one local recurrence [15]. In another cohort, 20% of juvenile PA patients who had undergone gross total resection experienced a radiologically observed regrowth [33]. Progression to higher grades is very rare and the vast majority of patients with chronic PA retain their WHO Grade I designation at regrowth [16]. However, they are likely to experience significant impacts on their quality of life from repeated operations and extended treatment courses.

As a chronic tumour affecting the patient over many years, one can hypothesise that the underlying biology of PA likely fluctuates over time. Many tumours demonstrate instability and evolve over time, for example by accumulating additional molecular abnormalities [44]. Repeated cycles of clonal selection may also produce marked cellular and genetic heterogeneity [19]. Heterogeneity encompasses not only the neoplastic component but extends to the tumour microenvironment, where microglia and macrophages are increasingly recognised for their interplay with tumour cells and contribution to tumour development and progression [22]. This is particularly relevant in PA, where microglia are suggested to play a role in tumour formation and ongoing support [12]. Moreover, microglial populations comprise as much as a third of all proliferating cells within PA, making it conceivable that chronic regrowth is partly driven by the microglial compartment [34]. Such changes to the cellular composition of PA could have implications for treatment. For example, it has recently been shown that microglial infiltration positively correlates with metrics predicting sensitivity to MAPK inhibition in low-grade glioma [52]. Therefore, an understanding of longitudinal tumour biology and cellular composition is essential for effective diagnosis, stratification, and management in the long term.

To our knowledge, aside from rare reports of malignant transformation, no detailed studies have investigated changes to the underlying biology or cellular composition of WHO Grade I PA over time. We have interrogated a cohort of WHO Grade I paediatric PA with patient-matched longitudinal sampling. We performed paired molecular analyses across time points, including bulk expression and methylation profiling, in conjunction with immunohistochemical validation. Subsequently, we demonstrate consistent changes to the tumour immune microenvironment that challenge the view of PA as a static entity.

## Methods

### Cohort selection

All tumours diagnosed as paediatric pilocytic astrocytoma over a 10-year period, within the BRAIN UK national tissue bank network, were identified. Subsequently, we retrieved all cases with multiple non-biopsy longitudinal samples from separate surgeries. Original diagnostic histological slides were reviewed by a neuropathologist (TSJ) to confirm the diagnosis of pilocytic astrocytoma WHO grade I by 2021 WHO criteria and to ensure the corresponding formalin-fixed paraffin-embedded (FFPE) tissue for analysis was representative of each tumour. All patients with an interval ≥6 months between operations plus adequate formalin-fixed paraffin-embedded tissue for both samples were included in the final cohort (*n* = 15). For cases with > 2 samples (*n* = 3), the earliest and latest samples were analysed, where facilitated by tissue availability. For control tissue in RNA sequencing and methylation profiling assays, temporal lobe tissue from patients who had undergone surgery for hippocampal sclerosis was used (*n* = 4). These were confirmed to be free of tumour and other structural pathology.

### Tissue preparation

FFPE blocks were trimmed with a microtome to 50 μm depth to remove tissue that was exposed to air and potential contamination. 10 μm thick rolls for DNA and RNA extraction were collected from freshly exposed block faces. Between samples, microtome blades were changed to avoid cross-contamination. Blades and cutting surfaces were cleaned with RNAseZap solution (ThermoFisher). For each case, 10 tissue rolls were taken. These were divided equally into two Eppendorf DNA LoBind 1.5 ml capped tubes: one for DNA, and another for RNA.

### DNA extraction

DNA was extracted from FFPE tissue using the Maxwell 16 FFPE Tissue LEV DNA Purification Kit (Promega) on a Maxwell 16 Research Instrument according to the manufacturer’s instructions. DNA for sequencing and methylation profiling were aliquoted separately. DNA for methylation profiling was bisulphite-converted using the Zymo EZ DNA Methylation-Gold Kit (Zymo Research). Bisulphite-converted DNA was treated using the Infinium FFPE DNA restore kit before assaying on the Illumina HumanMethylationEPIC BeadChip platform (Illumina).

### Methylation profiling

Illumina HumanMethylationEPIC BeadChip arrays were processed according to the Infinium HD Assay protocol. Processed arrays were scanned using an Illumina IScan to generate raw data as idat files. Analysis of methylation data was performed in R. Raw data were imported into R using the *minfi* package [3]. Detection p-values were generated for all probes and samples were excluded from further analysis if > 10% of probes demonstrated detection p-values > 0.01. To ensure the cohort did not include tumours with methylation data indicative of alternative diagnoses, the DKFZ MNP classifier was used for all samples [10]. Raw data were passed to MNP (versions 11b6; 12b6) for automatic processing. Classification scores < 0.7 were considered non-classifying (Additional File 1: Table S1). 22/30 were identified as PA, with 18 above the scoring threshold. None classified above this threshold as a non-PA diagnosis on either classifier version.

To confirm primary-longitudinal matches, beta values for 59 SNP probes included in the EPIC array were extracted. Correlations were calculated between samples in each pair. Pairs demonstrating Spearman correlation < 0.7 were excluded (*n* = 1). Next, data were normalised using the functional normalisation method within *minfi*. Probes mapping to X and Y chromosomes were excluded. Probes located within 50 bp of a SNP, probes known to cross-hybridise, and probes with a minor allele frequency > 5% were excluded. t-distributed stochastic neighbour embedding (tSNE), using the top 10,000 most variable probes across the cohort by median absolute deviation, was used to ensure tumours clustered away from control brain and remove pairs with outlier samples (*n* = 1). Identification of differentially methylated positions (DMP) was performed using *limma* [51]. Pairwise comparisons were made between matched primary and longitudinal samples from each patient. Pathway enrichment analysis of DMP lists was performed using *gometh* and *gsameth* functions in *missMethyl* [48].

Copy number analysis on EPIC array methylation data was performed in R using *conumee* [26]. Copy number changes were determined by manual inspection of individual copy number plots per sample, using LogR thresholds of + 0.1/-0.15 as guides for calling gains and losses.

### RNA extraction

RNA was extracted from FFPE tissue immediately after sectioning. Total RNA was extracted using Qiagen miRNeasy FFPE kits (Qiagen) according to the manufacturer’s instructions. RNA was eluted into 30 µl RNase-free water and stored at -80 °C. RNA quantity and integrity were assayed using a Qubit 2.0 (ThermoFisher) fluorometer and TapeStation 2200 (Agilent).

### RNA sequencing

cDNA was prepared from eluted RNA with the SuperScript IV RT kit (ThermoFisher), utilising random primers. Second-strand synthesis to create cDNA was carried out using the NEBNext Ultra II Non-Directional RNA Second Strand Synthesis Module (New England BioLabs). Library preparation was performed by the UCL Pathogen Genomics Unit using the SureSelectXT Low Input kit alongside Dual Index P5 Indexed Adaptors and the All Exon V6 + UTRs kit, according to the manufacturer’s instructions (Agilent). Sequencing was performed at the Institute of Cancer Research. Libraries were pooled and sequenced on the Illumina NovaSeq 6000 platform across 2 runs using NovaSeq 6000 V1.5 S1-200 Reagent kits (Illumina). To control for technical variation between runs, all 4 control samples were included on each run. Samples were sequenced with 2 × 75 bp paired-end reads at a target depth of 40–50 million paired-end reads per sample. Demultiplexing of sequenced data was performed by UCL Genomics.

Analysis of RNA sequencing data was performed in R. Raw fastq were processed using salmon (v1.3.0) to generate transcript-level quantifications against a partial transcriptome index constructed from the Ensembl hg38 Homo sapiens reference genome (release 100) [47]. For each sample, the total reads sequenced and percentage of reads that could be successfully mapped were recorded. Pairs were excluded from further analysis if either sample possessed < 10 million reads or > 50% of reads failed to map to the reference (*n* = 4). Quantified data passing these thresholds were read into R and condensed to gene-level data using *tximport* before conversion into a DESeq2-compatible dataset [39, 54]. Genes with < 10 combined counts across all samples were removed. Next, data were transformed by regularised log transformation and visualised with tSNE, using the top 5,000 most variably expressed genes across the cohort by median absolute deviation, to ensure that tumours clustered away from control brain and to identify outliers. The original un-transformed data were passed to DESeq2 for paired differential expression analysis of primary-longitudinal samples within each patient. Differentially expressed genes were considered significant at false discovery adjusted p-values < 0.1.

Ontogeny analysis of differentially expressed genes was performed using PANTHER (https://www.pantherdb.org/). Pre-ranked gene set enrichment analysis (GSEA) was performed using GSEA software (v4.1.0) [55]. Genes were ranked according to the Wald statistic assigned by DESeq2 during differential expression analysis then passed to GSEA for analysis against pre-built v7.4 hallmark, GO biological processes, and DESCARTES fetal cell type-specific gene sets, all hosted within the molecular signatures database (MSigDB) [9].

Gene set variation analysis (GSVA) was performed in R using the GSVA package [23]. The DESeq2 dataset previously described was normalised by variance stabilising transformation. GSVA was performed using the z-score method with a Gaussian kernel, against the DESCARTES fetal cell type gene sets detailed above.

To estimate immune cell proportions, gene-level expression data quantified in transcripts per million were uploaded to the CIBERSORT web portal (https://cibersortx.stanford.edu/) and assayed against LM22 leukocyte cell type gene signatures previously published by Newman et al. [45, 46]. CIBERSORT was run in absolute mode with B-mode batch correction. Quantile normalisation was disabled. Plotting of CIBERSORT output was performed in R.

### Targeted DNA sequencing

DNA was sequenced using a targeted, custom-designed capture panel (Agilent SureSelectXT; 5190 − 4862)(Additional File 1: Table S2). Library preparation and sequencing were performed at the Institute of Cancer Research. NGS libraries were prepared from 20-400ng DNA using the KAPA HyperPlus Kit (Roche) and IDT UDI 8 bp adapters (Integrated DNA Technologies) according to the manufacturer’s instructions. This included dual-SPRI size selection of libraries (250–500 bp). To optimise enrichment and reduce off-target capture, pooled, multiplexed, and amplified pre-capture libraries (12–13 samples per hybridisation) were hybridised overnight using 1 µg total DNA to custom-designed DNA baits complementary to genomic regions of interest. This was done using SureSelectXT Low Input Target Enrichment (Agilent). Hybridised DNA was PCR amplified and purified using AMPure XP beads (Beckman Coulter). Subsequently, DNA was quantified using the Qubit dsDNA High Sensitivity Assay Kit with the Qubit 3.0 fluorometer (Invitrogen) and High Sensitivity D1000 TapeStation (Agilent). Sequencing was performed on the Illumina NovaSeq 6000 platform (Illumina) using v1.5 chemistry according to the manufacturer’s instructions to output 150 bp paired-end reads. Demultiplexing was performed by UCL Genomics.

Sequencing data were analysed using the Royal Marsden NHS Foundation Trust DNA panel pipeline (MDIMSv4). Reads were aligned to the GRCh37/hg19 genome using BWA-MEM (v0.7.17). QC metrics and PCR duplicate flagging were performed by Picard (v2.23.8). GATK (v4.1.9.0) was used for realigning around indels and base quality score recalibration. GATK-Mutect2 was used for variant calling. Manta (v1.2.2) and Pindel (v0.2.5b8) were used for structural variant detection. Filtering was performed using dbSNP build 153 to remove variants with a population allele frequency > 1%. A panel of normal (PON) comprised of fetal control tissue (*n* = 30) was concurrently sequenced (Additional File 1: Table S3). No material with known karyotypic or developmental abnormalities was included within the PON. Somatic variants within the PON were screened using Mutect2 in tumour-only mode. Structural and single nucleotide variants identified within tumours were excluded if present in ≥ 3 PON or if located within regions demonstrating poor sequencing quality in PON. Synonymous and non-coding single nucleotide variants were filtered out from VEP (v103) annotated VCF files. Single nucleotide variants were excluded if detected at < 5% variant allele frequency, if < 5 reads supported the variant, or if total coverage of the region was < 20 reads. Exclusion criteria were waived if an identical variant, exceeding these criteria, was detected in the matched sample. All single nucleotide and structural variants were confirmed manually in IGV (v2.16.2). Classification and pathogenicity prediction were performed using Franklin (https://franklin.genoox.com/).

### Immunohistochemistry

5 μm thick sections were taken from FFPE blocks previously used for DNA and RNA extractions. Immunohistochemistry was performed against CD68 (1:250, Agilent, M0876), IBA1 (1:10,000, Abcam, ab178846), CD163 (Pre-diluted, CellMarque, 163 M-17), TREM2 (1:100, Cell Signaling, 91068), and CD8 (Pre-diluted, Leica, PA0183). All immunohistochemistry was performed using a Ventana BenchMark ULTRA (Roche) or Leica BondMax machine (Leica Biosystems).

Immunohistochemical quantification was performed using QuPath (v0.4.4) [5]. Whole slide images were generated using a Leica Aperio CS2 scanner (Leica Biosystems). Immunohistochemical stains were quantified as a percentage of positively stained cells versus total cells detected. Regions with excess background signal, staining artefacts, large blood vessels, and areas with extensive haemorrhage were excluded. Staining vectors used for colour deconvolution were automatically estimated for each slide using a subsampled region containing background staining plus true positive and negative signals. Statistical analysis of immunohistochemical quantification was performed in a paired manner between matched primary and longitudinal samples from each patient.

## Results

### Cohort characteristics

After selecting cases with longitudinal sampling across multiple operations at least 6 months apart, we identified 15 cases with paired primary-longitudinal samples and sufficient FFPE material for DNA and RNA extractions (Table 1, Additional File 1: Table S4). These were 9 males and 6 females, with a mean age at diagnosis of 69.3 months (10–150 months). The mean follow-up time was 101 months (4-194 months) and 13/15 patients (87%) were alive at last follow-up. One patient died from disease, and another died of other causes. The most frequent tumour location was the cerebellum (*n* = 6), followed by the optic pathway (*n* = 3), brainstem (*n* = 3), temporal lobe (*n* = 1), thalamus (*n* = 1), and thoracic spine (*n* = 1). 9 Patients possessed *KIAA1549::BRAF* fusions. One patient possessed a *BRAF* V600E variant and one patient possessed an *NF1* variant. All tumours in the cohort were sporadic except for the single NF1-associated case. The mean interval between primary and longitudinal samples was 2.73 years (0.5-8 years).

**Table 1:** Summary of the cohort clinical information and *BRAF/NF1* alterations

Cohort	Age at diagnosis (Months)	Sample interval (Years)	BRAF/NF1 alterations
All (*n* = 15)	69.3 (10–150)	2.73 (0.5-8)	*KIAA1549::BRAF* (*n* = 9)
			*BRAF* V600E (*n* = 1)
			*NF1* deletion (*n* = 1)
Male (*n* = 9)	72.9 (11–150)	2.8 (0.5-4)	*KIAA1549::BRAF* (*n* = 4)
			*BRAF* V600E (*n* = 1)
			*NF1* deletion (*n* = 1)
Female (*n* = 6)	63.8 (10–146)	2.5 (0.5-8)	*KIAA1549::BRAF* (*n* = 5)

### Pilocytic astrocytomas demonstrate immune-related changes in methylation and expression over time

**Fig. 1:**
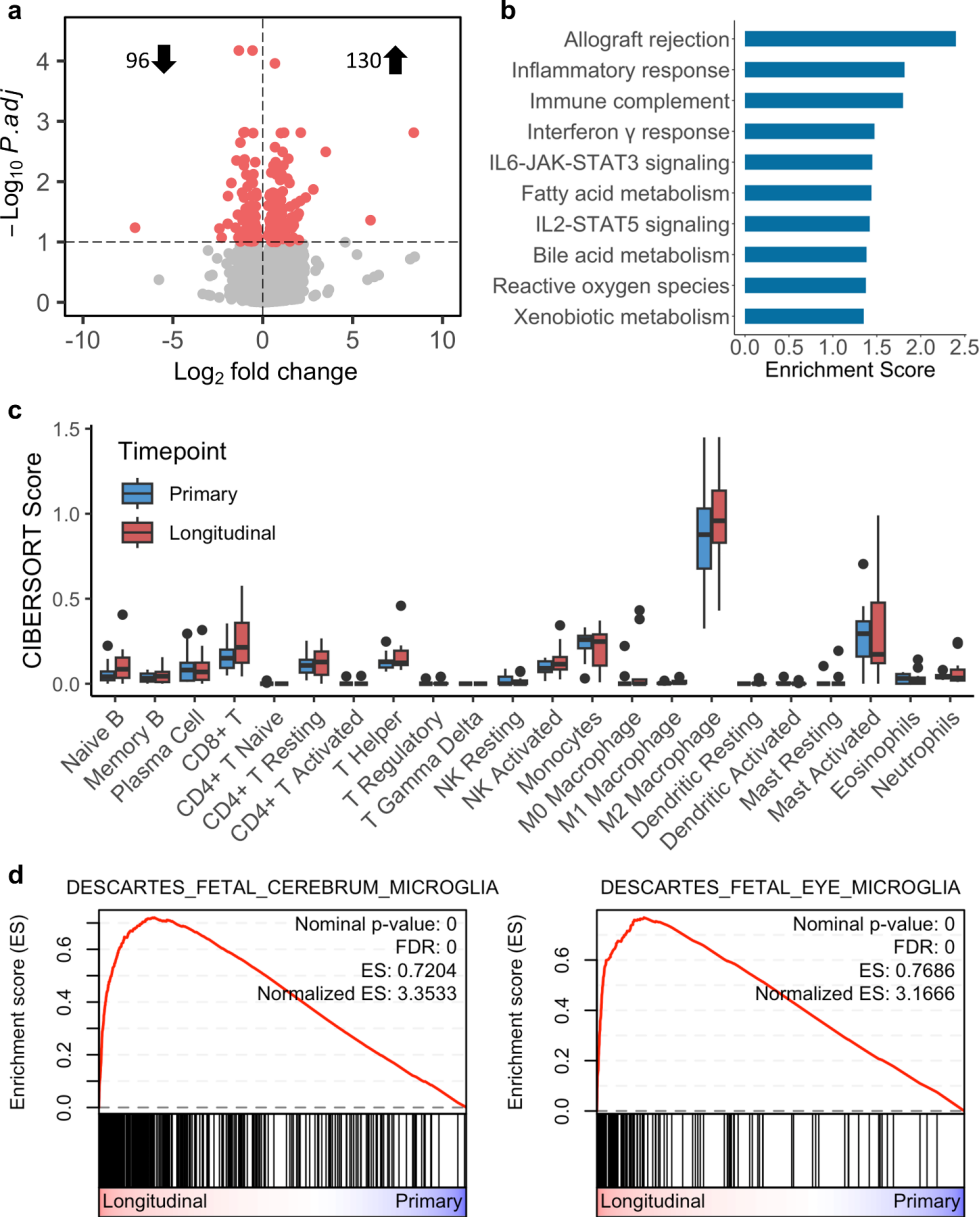
Longitudinal samples from pilocytic astrocytoma are enriched for microglial expression signatures. Paired differential expression analysis identifies 226 differentially expressed genes between primary and longitudinal pilocytic astrocytoma (**a**). Gene set enrichment analysis of expression data indicates expression changes correspond to enrichments for immune-related processes (**b**). CIBERSORT deconvolution of expression data against leukocyte signatures highlights an M2 macrophage signature as the predominant pilocytic astrocytoma immune component (**c**). Subsequently, gene set enrichment analysis against fetal cell type gene sets demonstrates an enrichment in longitudinal samples for microglial gene sets, suggesting pilocytic astrocytoma experience an increase in microglial content over time (**d**). ES = enrichment score

To investigate changes in the expression and methylation profiles of PA over time, we performed RNA sequencing and methylation array profiling. After quality control, we were able to analyse expression data for 11/15 and methylation data for 13/15 pairs. For both methylation and expression data, tumours clustered together away from controls on tSNE (Additional File 2: Figure S1). We subsequently performed pairwise differential expression and methylation assays between primary and longitudinal samples. By RNASeq, we identified 226 differentially expressed genes at adjusted p-values < 0.1 (Fig. 1a, Additional File 1: Table S5). The majority were upregulated in longitudinal samples (*n* = 130). Ontogeny analysis suggested an over-representation of genes involved in immune and inflammatory responses (Additional File 1: Table S6). These enrichments were further confirmed by Gene Set Enrichment Analysis (GSEA) of expression data against the MSigDB hallmark and GO biological processes datasets (Table 2). 11 hallmark gene sets were enriched in longitudinal samples (Additional File 1: Table S7). Among these, the strongest enrichments were for immune-related pathways (Fig. 1b). Complementing this, when we assayed cytokine-related pathways by GSEA using the GO biological processes dataset we identified 16 pathways enriched in longitudinal samples that were positively associated with cytokine regulation, production, and response (Additional File 1: Table S8).

**Table 2:** Longitudinal gene set enrichments

Category	Gene Set	NES	q-val
Hallmark	Allograft rejection	2.399	0.000
Hallmark	Inflammatory response	1.815	0.001
Hallmark	Complement	1.798	0.001
Hallmark	Interferon gamma response	1.472	0.051
Hallmark	IL6 JAK STAT3 signalling	1.448	0.056
GO Cytokines	Cytokine production involved in inflammatory response	2.033	0.001
GO Cytokines	Negative regulation of cytokine production	2.012	0.001
GO Cytokines	Positive regulation of cytokine production	1.965	0.002
GO Cytokines	Cytokine production involved in immune response	1.958	0.002
GO Cytokines	Positive regulation of cytokine production in inflammatory response	1.927	0.003
Fetal Cell Type	Descartes fetal cerebrum microglia	3.353	0.000
Fetal Cell Type	Descartes fetal eye microglia	3.167	0.000
Fetal Cell Type	Descartes fetal lung myeloid cells	3.099	0.000
Fetal Cell Type	Descartes fetal pancreas myeloid cells	3.063	0.000
Fetal Cell Type	Descartes fetal intestine myeloid cells	3.022	0.000

**Legend**: Top gene sets enriched in longitudinal samples for MSigDB hallmark, GO cytokine, and fetal cell type gene set categories. NES = normalised enrichment score

Contrasting the expression data, while we observed 28,130 differentially methylated CpGs (15,348 hyper-, 12,782 hypomethylated) by methylation profiling, none retained significance after false discovery adjustment. However, KEGG enrichment analysis of CpGs differentially methylated at a standard p-value < 0.05 indicated over-representations of Ras signalling and several pathways involved in cell migration. Moreover, analysis of CpGs that were hypomethylated in longitudinal samples revealed an over-representation of immune and inflammatory-related pathways, mirroring our expression data. (Additional File 1: Table S9).

Combined, these data highlight consistent changes to gene expression in PA over time, favouring immune-related functions. These changes are mirrored by complementary hypomethylation of genes involved in immune-related pathways. It was these changes which we focused on during downstream analyses.

### Microglial phenotypes become enriched in pilocytic astrocytoma over time

We hypothesised that immune-related overrepresentations could be explained by changes over time to the immune and cellular composition of the tumours. To estimate the composition of major immune cell phenotypes within our samples, we performed CIBERSORT analysis on the expression data. This demonstrated a striking predominance of an M2 macrophage signature across both primary and longitudinal samples (Fig. 1c). In the context of the CNS, this dominance led us to question whether changes in the abundance of microglial populations explained immune-related expression changes. To test this, we performed GSEA on our expression data against cell-type specific gene sets generated from single-cell analyses of fetal tissues [9]. These gene sets included neural cell and microglial signatures. We identified strong enrichments for myeloid lineages, particularly microglial gene sets, within longitudinal samples (Table 2; Fig. 1d, Additional File 1: Table S10). In contrast, primary samples displayed an enrichment for oligodendrocyte and neuronal-associated gene sets.

**Fig. 2:**
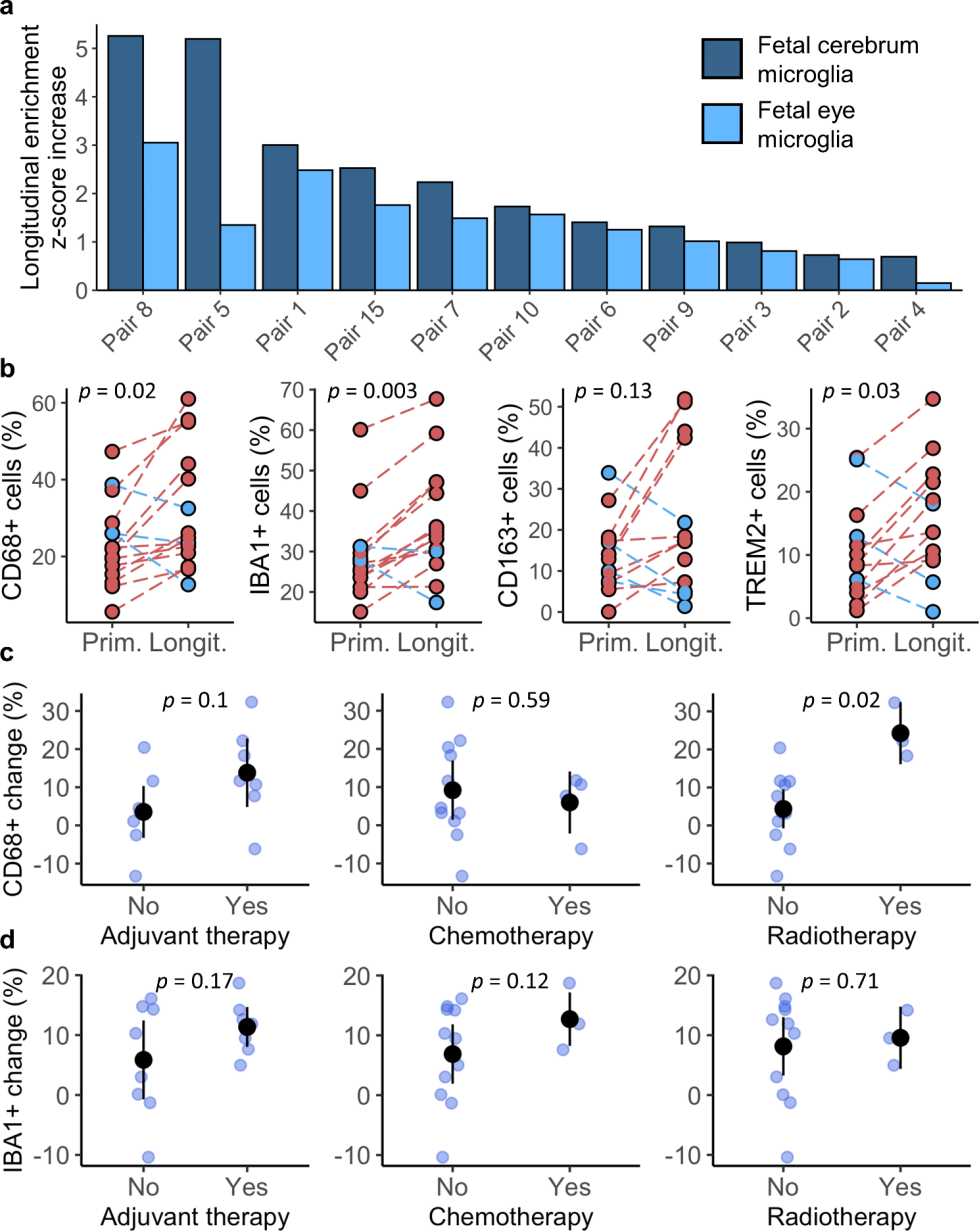
Microglial enrichment is consistent across pilocytic astrocytoma, can be quantified immunohistochemically, and includes anti-inflammatory phenotypes. When longitudinal changes in enrichment scores for microglial gene sets per patient are assessed by gene set variation analysis, all patients with expression data (*n* = 11) demonstrate an increase over time (**a**). Subsequent immunohistochemistry demonstrates longitudinal increases in myeloid/microglial markers CD68 and IBA1, which are concurrent with rises in M2-like/anti-inflammatory markers CD163 and TREM2 (**b**). Microglial enrichments appear only partially related to treatment modality. When patients are stratified according to treatment received at any timepoint between samples, a larger increase in CD68+ cells is associated with receipt of radiotherapy (**c**). However, this effect was not replicated for IBA1+ cell increases (**d**)

As GSEA is a group-level analysis, we used gene set variation analysis (GSVA) to assay expression profiles on a sample-by-sample basis. We hypothesised that this would determine whether cell type enrichments were present in all cases or whether this effect was restricted to a subset. After performing GSVA against fetal cell type-specific gene sets, we confirmed enrichments for two gene sets in longitudinal samples compared to their respective primaries (Fig. 2a). These were fetal cerebral microglia and fetal eye microglia. Strikingly, we observed increases in microglial enrichment over time in all individuals, demonstrating a consistent effect across the cohort.

Methylation is a negative regulator of expression, so we hypothesised that upregulation of microglial gene sets should coincide with decreased methylation of corresponding CpGs and reviewed our methylation data to identify complementary changes to methylation. Overrepresentation analyses of hyper- and hypomethylated CpG subsets against cell type-specific gene sets demonstrated enrichments for microglial and myeloid signatures in hypomethylated CpGs, complementary to expression enrichments (Additional File 1: Table S11). The strongest enrichments were fetal cerebellum microglia, fetal microglia, and fetal eye microglia. Conversely, among hypermethylated CpGs, we identified no cell type-specific enrichments.

Taken together, these data confirm the immune-related changes we previously observed and indicate increased microglial expression phenotypes in PA over time. This is supported by complementary methylation patterns as microglial-related gene sets demonstrate concurrent hypomethylation.

### Immunohistochemistry confirms microglial enrichment over time alongside elevated M2-like markers

To validate longitudinal microglial enrichments in expression and methylation profiles we performed immunohistochemistry against two markers expressed by microglia, CD68 and IBA1, on primary and longitudinal sections from all 15 PA pairs (Fig. 2b, Additional File 1: Table S12). Representative staining for CD68 and IBA1 are shown in Additional File 2: Figure S2. After digital quantification of staining, we identified significant longitudinal increases in the proportions of CD68 (*p* < 0.05) and IBA1 (*p* < 0.005) immunoreactive cells as a percentage of the total cells detected on each slide. 12/15 (80%) patients demonstrated an increase in the proportion of CD68+ cells over time, while 13/15 (87%) patients showed increasing proportions of IBA1+ cells. Total percentages of CD68+ and IBA1+ cells correlated strongly with one another (*R* = 0.65, *p* < 0.005). CD68+ and IBA1+ content also correlated strongly with enrichment scores for microglial gene sets from our GSVA analysis (*R* > 0.7, *p* < 0.001 in all comparisons) (Additional File 2: Figure S2). Across the cohort, including pairs with negative change, CD68+ cells increased as a proportion of total cell content by an average of 8.3% between primary and longitudinal samples. Likewise, IBA1+ cells increased by an average of 8.4%. These data validate and extend expression and methylation findings that the majority of PA in our cohort transition towards increased microglial presence over time.

Glioma-associated microglia frequently acquire an M2-like phenotype and we had previously noted abundant M2 macrophage signatures by CIBERSORT [11, 37]. We hypothesised that microglial increases may represent an enrichment for M2-like or anti-inflammatory microglia. To test this, we performed immunohistochemistry against CD163, a putative M2-polarisation marker and TREM2, a protein associated with immunosuppressive activity in tumour-associated microglia/macrophages (Fig. 2b, Additional File 1: Table S12) [38, 40, 43]. Representative staining for CD163 and TREM2 are shown in Additional File 2: Figure S2. Immunohistochemistry was completed for primary and longitudinal samples in 13/15 pairs, as 2 lacked sufficient remaining tissue for analysis in their longitudinal samples. For CD163, we observed increases over time in 9/13 pairs with a mean increase, including pairs with negative change, of 8.8% as a proportion of total cell content (*p* = 0.13). For TREM2, 10/13 pairs increased longitudinally with a mean increase of 5.3% (*p* < 0.05). These data suggest microglial enrichment is at least partly driven by expansion or recruitment of microglia with an immunosuppressive or M2-like phenotype.

While microglial signatures were the dominant signal in our initial analyses, among differentially expressed genes identified in our expression analysis we also noticed several T cell markers including CD6, CD109, CD48, and CD8. CD8+ T cells have previously been reported as the second largest immune cell component of PA after microglia, and in LGG are suggested to work with microglia to promote a tumour-supportive environment [13, 20]. This prompted us to perform immunohistochemistry against CD8. However, CD8 immunoreactivity was consistently low across the cohort, averaging 1.9% (0-6.8%) of total cells (Additional File 1: Table S12), with no significant differences between primary and longitudinal samples, indicating this immune population does not increase alongside microglia.

### Microglial enrichments are not explained by clinical characteristics and only poorly correlate with therapy

Microglia are purported to be important regulators of PA initiation and growth and comprise a substantial fraction of proliferative cells [12, 21, 34]. However, a range of clinical characteristics are potential confounders to our observation of microglial enrichment over time. We sought to assess the relationship between these and our immunohistochemical data. These features included NF1 status, treatment modality, receipt of corticosteroids before surgery, patient age, sex, and tumour location (Additional File 1: Table S13).

NF1-associated PA are associated with increased microglia versus sporadic tumours [53]. To confirm this was not the driving factor behind CD68+/IBA1+ cell increases, we repeated the analysis of our immunohistochemical data after excluding the single patient with a known *NF1* variant (Additional File 2: Figure S3). Longitudinal increases in CD68 (*p* < 0.05) and IBA1 (*p* < 0.005) immunoreactive cells retained significance, indicating that NF1 status does not explain microglial enrichment over time.

Next, we hypothesised that changes to microglial populations could be impacted by whether patients received adjuvant chemo- or radiotherapy between operations. Here, we compared patients by the size of the change in the percentage of CD68/IBA1 immunoreactive cells between their respective primary/longitudinal samples. Initially, we stratified patients according to therapy in the 6 months preceding their longitudinal operation. However, too few patients received therapy within this window (*n* = 3/15) for meaningful analysis. Therefore, we expanded the range to include therapy at any time between samples (*n* = 7/15). This expansion resulted in mean treatment timings of 11 (1–27) and 16 (1–30) months before longitudinal sampling for chemotherapy and radiotherapy, respectively. For CD68 we observed that patients receiving any adjuvant therapy between samples trended towards greater increases in CD68+ cell population, though this was not statistically significant (*p* = 0.1). When categorised into chemo- and radiotherapy, this appeared to be driven by radiotherapy (*p* < 0.05), rather than chemotherapy (*p* = 0.59)(Fig. 2c). Conversely, for IBA1 we did not identify a significant relationship with adjuvant therapy, chemotherapy, or radiotherapy (Fig. 2d). As the only significant trend related to a single marker and treatment modality, it is unlikely that treatment sufficiently explains microglial enrichment over time.

We also questioned whether receipt of corticosteroids in the pre-operative period was related to microglial content, due to their anti-inflammatory effect. Patients were grouped according to receipt of corticosteroids in the period up to 6 months before their operation. As we lacked precise timing for treatments given intra-operatively, patients receiving intra-operative corticosteroids were grouped alongside untreated. We found no significant relationship between receipt of corticosteroids and CD68+ or IBA1+ cell content across all primary and longitudinal samples (Additional File 2: Figure S3). Similarly, when patients were stratified according to the timing of corticosteroids (e.g., receipt of corticosteroids at either primary or longitudinal timepoint but not the other) we found no effect on the size of the change in CD68/IBA1 immunoreactive cells between sample pairs.

Finally, we assessed the impact of age at surgery, sampling interval, sex, and tumour location. For age and sampling interval, we hypothesised microglial enrichment may be a function of patient age or may represent inflammation from short intervals between operations. However, we found no significant correlation between age at surgery and CD68+ or IBA1+ cell content. (Additional File 2: Figure S4). Nor did we observe any significant relationship between the size or direction of the change in CD68+ or IBA1+ cell content and the duration of the interval between operations, suggesting microglial enrichment is not explained by inflammation over shorter periods. For sex, we noted borderline non-significant trends where male patients had higher percentages of CD68+ cells in primary (*p* = 0.11) and longitudinal (*p* = 0.07) samples (Additional File 2: Figure S4). However, these were not reproduced for IBA1. Additionally, we found no association between sex and longitudinal changes in CD68/IBA1. To address tumour location, we compared tumours at two levels (Additional File 2: Figure S5). Firstly, we excluded locations with *n* = 1 (temporal, thalamic, spine) and compared the remaining locations (brainstem cerebellum, optic pathway). The only trends identified were for optic pathway tumours possessing larger CD68+ populations at the longitudinal time point compared to cerebellar, but not brain stem tumours (*p* = 0.05), while brain stem tumours experienced larger increases over time in CD68 than cerebellar, but not optic pathway tumours (*p* = 0.03). Both were weak trends, and brain stem and optic pathway locations had small sample sizes of 3 with significant variability in quantification data, heavily limiting the interpretability of these tests. Subsequently, we grouped tumours into supra- and infratentorial locations, excluding the single spinal PA. Here, the only trend we identified was that supratentorial tumours possessed more CD68+ cells than infratentorial tumours longitudinally (*p* < 0.01). Infra-/supratentorial location had no impact on the size of CD68+ or IBA1+ change over time.

### Pilocytic astrocytomas possess varied genetic alterations but do not acquire additional drivers

**Fig. 3:**
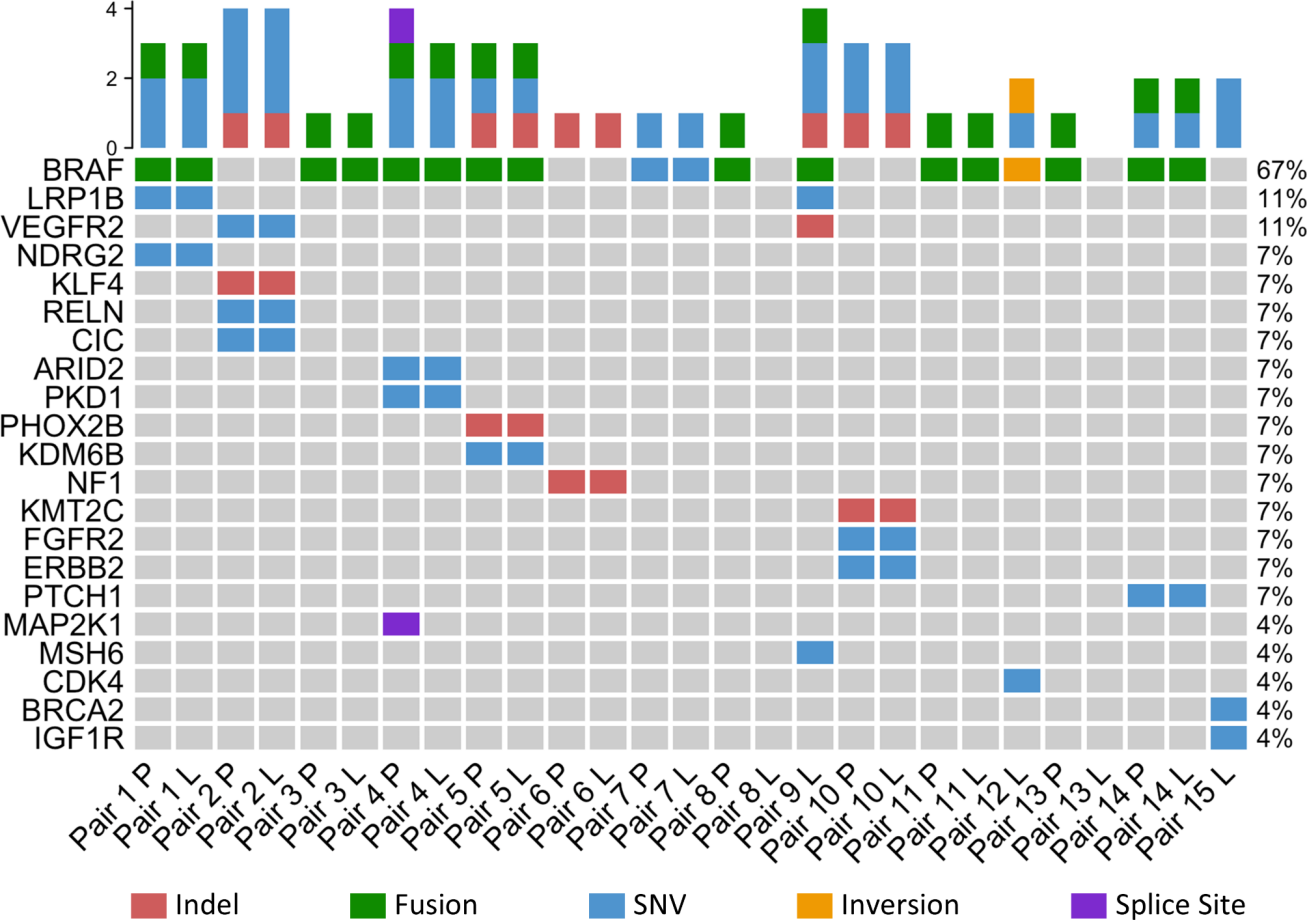
Pilocytic astrocytoma do not routinely acquire additional alterations. Targeted DNA panel sequencing demonstrates no consistent changes over time to the mutational profiles between primary and longitudinal pilocytic astrocytoma

Having demonstrated changes to the gene expression, epigenetic, and microglial components of PA over time, we hypothesised we may observe increased heterogeneity at the DNA level via the accumulation of additional alterations. To test this, we performed targeted DNA sequencing against a panel of paediatric CNS tumour-relevant genes. After DNA quality control, we sequenced primary and longitudinal samples for 12/15 pairs (Fig. 3, Additional File 1: Table S14). Among these, we identified pathogenic *BRAF* alterations in 9 pairs (8 *KIAA1549::BRAF*, 1 *BRAF* V600E). In the remaining 3 complete pairs, we observed alterations in MAPK/ERK-relevant genes including *NF1* in one sample, *FGFR2* and *ERBB2* in another, and *KLF4* plus *VEGFR2* in the third. Comparing matched primary/longitudinal samples, there were no consistent changes over time. 8/12 pairs demonstrated identical variants between samples. Of the remaining 4 pairs, 2 had detectable *KIAA1549::BRAF* fusions in the primary sample but no detectable variants in the longitudinal, likely due to poor sequencing depth. One tumour demonstrated a *MAP2K1* alteration in the primary that was not replicated in the longitudinal sample. The final complete pair possessed a *DEPDC5* variant in the longitudinal sample that was not present in the primary.

In 3/15 pairs, we could only assay the longitudinal sample. One tumour possessed a *KIAA1549::BRAF* fusion alongside *VEGFR2*, *MSH6*, and *LRP1B* variants. Another tumour demonstrated partial *KIAA1549* inversion in addition to *CDK4* and *KDM6B* variants. The last lone longitudinal sample harboured a *BRCA2* variant alongside an *IGF1R* variant. Overall, we identified variants in canonical PA-associated genes, or genes relevant to MAPK/ERK activity, in almost all cases. However, these data suggest that across these timespans, PA do not routinely acquire additional somatic variants. Additionally, we saw no impact for genotype on the microglial content within our cohort except for the single NF1-associated tumour, which possessed the greatest overall proportion of IBA1+ cells across both samples.

We also inferred copy number profiles from methylation data for all sample pairs that passed quality control (*n* = 13) (Additional File 1: Table S15). By manual inspection of individual profiles, we observed no robust copy number abnormalities at either time point in 10/13 (77%) patients. One patient’s tumour had gains affecting chromosomes 5, 6, 11, and 20 which were present in both samples. In another, we observed a gain affecting chromosome 7, which was only present in the longitudinal sample. Lastly, in one patient we could not identify any copy number abnormalities in the primary sample and the profile for the longitudinal sample was of insufficient quality for assessment.

## Discussion

Pilocytic astrocytomas are often approached as a static tumour, but many patients suffer chronic disease and regrowth [2, 15, 18, 33]. Cancers evolve over time, for example via genomic instability and accumulation of molecular aberrations [44]. They may also undergo repeated cycles of clonal selection, producing marked cellular and genetic heterogeneity [19]. It is therefore reasonable to expect that pilocytic astrocytomas possess the capacity for gradual evolution, an understanding of which is desirable for monitoring, stratification, and long-term management. However, no studies have addressed biological change over time in these common tumours.

We used molecular profiling to assess the pathological stability of PA with matched primary and longitudinal samples. These represent all available tumours diagnosed over a 10-year period at a major UK paediatric brain tumour centre with matched tissue at multiple time points. We detected differences over time in expression and methylation profiles indicating changes to the immune microenvironment, with increases in microglial cell content occurring in longitudinal samples relative to primary tumours. We validated this immunohistochemically with CD68 and IBA1, and corresponding increases in CD163 and TREM2 suggest large parts of these enrichments are driven by M2-like or immunosuppressive myeloid cells.

Existing data indicate microglia play a crucial role in the development and maintenance of pilocytic astrocytoma, which can be distinguished from other tumours by their high microglial content and proliferation of microglia relative to tumour cells [34]. Studies in mice indicate that while a *KIAA1549::BRAF* fusion is sufficient to generate glioma-like lesions, this is associated with increased Iba1+ microglia infiltration and is inhibited if Ccr2-mediated microglial recruitment is blocked [12, 32]. Moreover, it is proposed that permissive stromal signals from the microglial component are required to overcome *KIAA1549::BRAF* mediated senescence (reviewed in [21]). This suggests microglia foster a pro-tumorigenic microenvironment at formation and promote tumour maintenance throughout the lifespan of PA. Interestingly, microglial infiltration has recently been shown to positively correlate with sensitivity to therapy via MAPK inhibition, highlighting the potential importance of these cells for treatment response [52]. In this context, while the exact role of our observed microglial increase is unclear, several hypotheses are possible.

One potential conclusion is that microglial accumulation occurs over the natural course of PA evolution, with the same mechanisms that initially attract microglia at tumorigenesis continuing to concentrate them over time. This may be MAPK-driven, as increasing accumulation of CD68+ and CD163+ microglia is similarly observed in melanoma, correlating with greater tumour size and Breslow depth, and at significantly higher levels in *BRAF*-altered samples [57]. Microglia are known to accumulate in glioma, and an attractive mechanism could explain strikingly larger microglial populations in PA versus other astrocytic tumours [11, 22, 34]. Moreover, *BRAF* mutant paediatric low-grade glioma cells in vitro secrete microglia-recruiting cytokines [35]. In the context of regrowth, microglia may be recruited by remnant tumour cells following resection via a range of chemoattractants [22]. Recruited microglia could rapidly repopulate the tumour niche in comparison to tumour cells, which are mainly thought to be senescent, potentially explaining the increase in microglia as a proportion of cells over time [8].

Microglia recruited by glioma are reported to acquire M2-like or mixed M1/M2-like phenotypes, thus the expression of anti-inflammatory markers CD163 and TREM2 by large proportions of PA-associated microglia supports the idea that these represent recruited microglia [11, 37, 38, 43, 58, 61]. In most of our cohort, longitudinal CD163+ and TREM2+ enrichments indicated large fractions of observed microglia are not part of a pro-inflammatory response. CD163+ microglia have previously been reported in large proportions in PA, similarly corresponding in number to around half the IBA1+ population [61]. To our knowledge, TREM2 expression has not been investigated in PA. However, in other malignancies including glioma, it is noted as a negative prognostic marker and is thought to modulate anti-tumour immune responses through immunosuppressive activity, further supporting the hypothesis that these represent microglia co-opted into a supportive role [31, 41, 43, 56]. If confirmed, this activity presents a potential future therapeutic target, for example via pro-inflammatory re-programming of microglia.

Microglial enrichment was not adequately explained by a range of potential confounders, and none significantly associated with the microglia-specific marker IBA1. The strongest relationship was a weak correlation with adjuvant therapy; a trend lacking robustness that applied only to radiotherapy – which only 3 patients received between operations – and was limited to CD68+ cells. As CD68 is strongly upregulated during inflammation and is expressed by other myeloid lineages, including infiltrating macrophages which are recruited after irradiation, this may reflect a broader inflammatory response separate from the increase in M2-like microglia, which when combined present a mixed phenotype [14, 25, 42]. In contrast, IBA1 is restricted to microglia and while expression elevates during microglial activation it does not necessarily represent an activated subset; rather characterising all microglia and demonstrating smaller fluctuations relative to other inflammatory markers across microglial states [24, 27, 28, 60].

## Conclusions

In summary, our findings challenge the view of pilocytic astrocytoma as a static entity, demonstrating an enrichment for microglia over time which seems to harbour M2-like or immunosuppressive fractions. There are a few limitations to the data which require caution. These include generalisability due to the cohort size and the possibility that pilocytic astrocytoma that require multiple operations, such as those in our cohort, may differ biologically from those which are manageable in the long-term without further resection. Nevertheless, the finding that the tumour microenvironment appears to shift over time in these common tumours presents a novel dynamic in our understanding of pilocytic astrocytoma biology. The driver for this is unclear and due to our cohort size plus the limitations of the available FFPE material, we are unable to elucidate a causative mechanism. However, given the increasingly recognised importance of the immune microenvironment in tumour biology, further characterisation of microglial activity in PA is warranted to unveil their interplay with the tumour. This is particularly timely given the current therapeutic landscape for PA and MAPK-driven low-grade glioma, in which MEK/RAF inhibitors are the subject of trials and are close to becoming first-line therapy [6, 7, 59]. In addition to their anti-tumour effects, these agents have immunomodulatory properties and in other cancers are reported to increase anti-tumour immunity, decrease immunosuppressive activity, and inhibit the accumulation of tumour-associated macrophages [4, 17, 50]. In low-grade glioma specifically, immune infiltration has recently been reported to affect sensitivity to treatment with MAPK inhibitors [52]. Taken alongside our data suggesting the microglial population fluctuates over time as part of the tumour’s natural course, an evaluation of immune infiltration during PA assessment may be valuable as it could influence response to targeted therapy. Likewise, though we have not investigated it here, radiological studies of longitudinal tumours may allow for ongoing non-invasive monitoring of immune infiltration and potentially highlight longitudinal imaging changes. Though our data cannot define precise parameters for the stratification of tumours based on their immune component, our observation of the temporal fluidity of the tumour immune microenvironment raises the question of whether an optimal timing or immune state exists for the delivery of targeted treatments that may interact with the immune milieu. Further study of the immune microenvironment in pilocytic astrocytoma therefore appears likely to highlight strategies for improving the long-term management of these common tumours.

## Electronic supplementary material

Below is the link to the electronic supplementary material.

Supplementary Material 1

Supplementary Material 2

## Data Availability

The data that support the findings of this study are available on reasonable request from the corresponding author. R scripts used for the analysis of methylation data, RNA sequencing data, statistical analysis, and production of plots are available at https://github.com/tj-stone/longitudinal_pa.

## Abbreviations

DMP: Differentially methylated position
FFPE: Formalin-fixed paraffin-embedded
GSEA: Gene Set Enrichment Analysis
GSVA: Gene Set Variation Analysis
MSigDB: Molecular Signatures Database
PA: Pilocytic astrocytoma
PON: Panel of normals
tSNE: t-distributed stochastic neighbour embedding
