# Supplementary material for: The tumour microenvironment of pilocytic astrocytoma evolves over time via enrichment for microglia
Source: Acta Neuropathol Commun. 2025 Feb 13;13:30. doi: 10.1186/s40478-024-01922-9 (PMC11823165; doi:10.1186/s40478-024-01922-9)
Supplement: Supplementary file 1 — Supplementary Material 1 [file 40478_2024_1922_MOESM1_ESM.pdf]

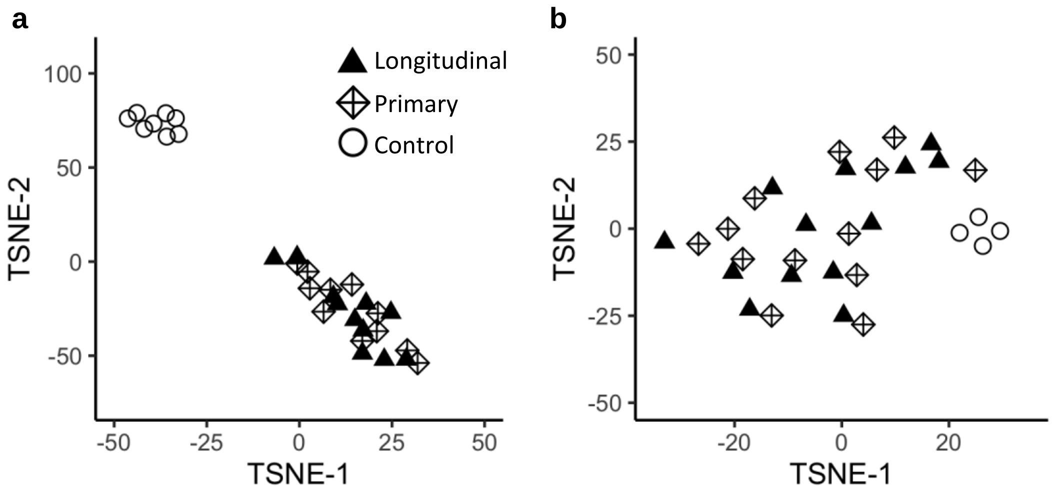

Supplementary Figure S1. tSNE plots for RNA sequencing (**a**) and Illumina EPIC array methylation (**b**) demonstrate separation of tumours from controls.

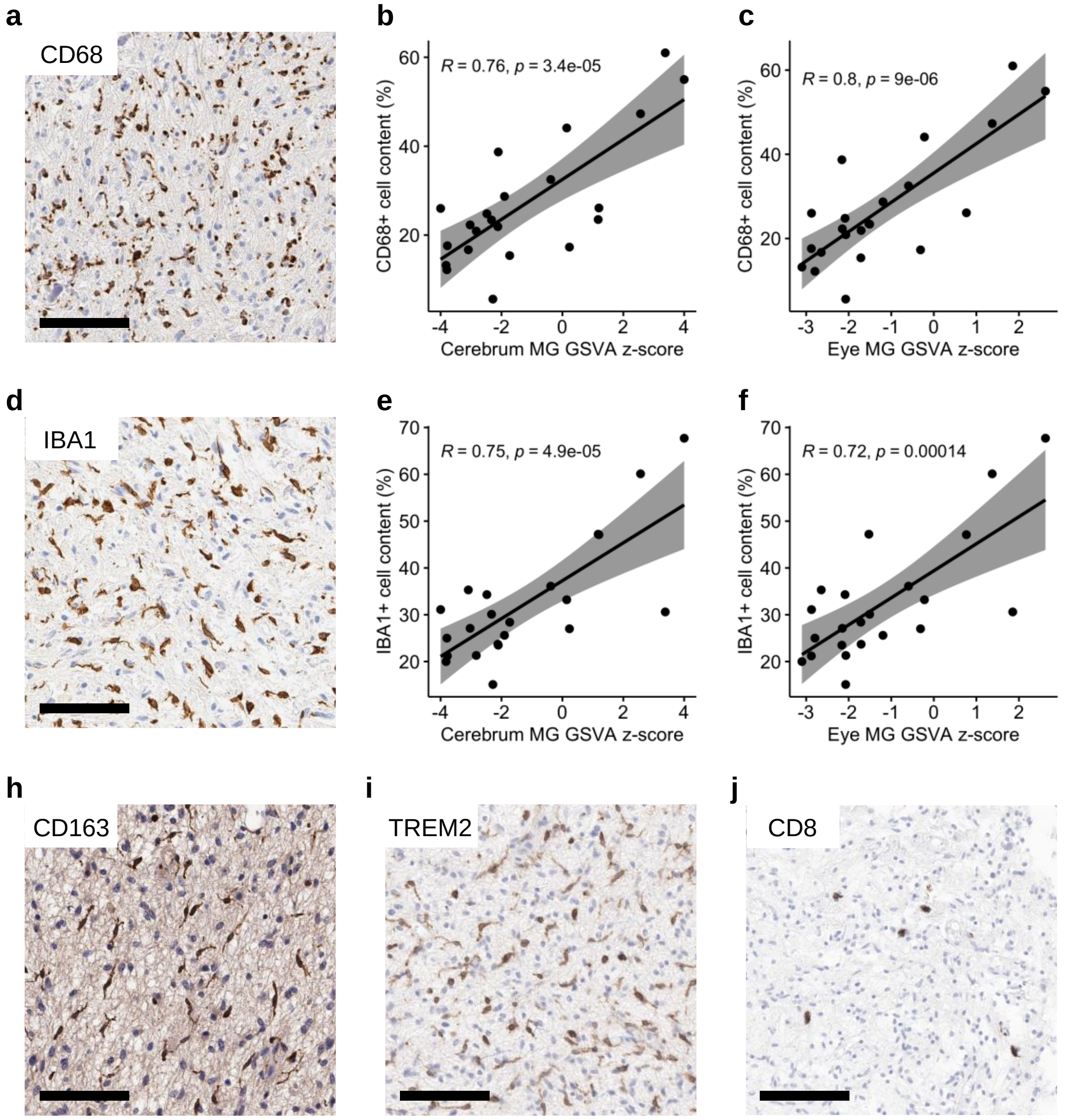

Supplementary Figure S2. Representative immunohistochemical stains for CD68, IBA1, CD163, TREM2, and CD8 (**a**, **d**, **h**, **I**, **j**). CD68 and IBA1 content strongly correlate with microglial enrichment z-scores in expression data as determined by gene set variation analysis (**b**, **c**, **e**, **f**). Scale bars 100µm.

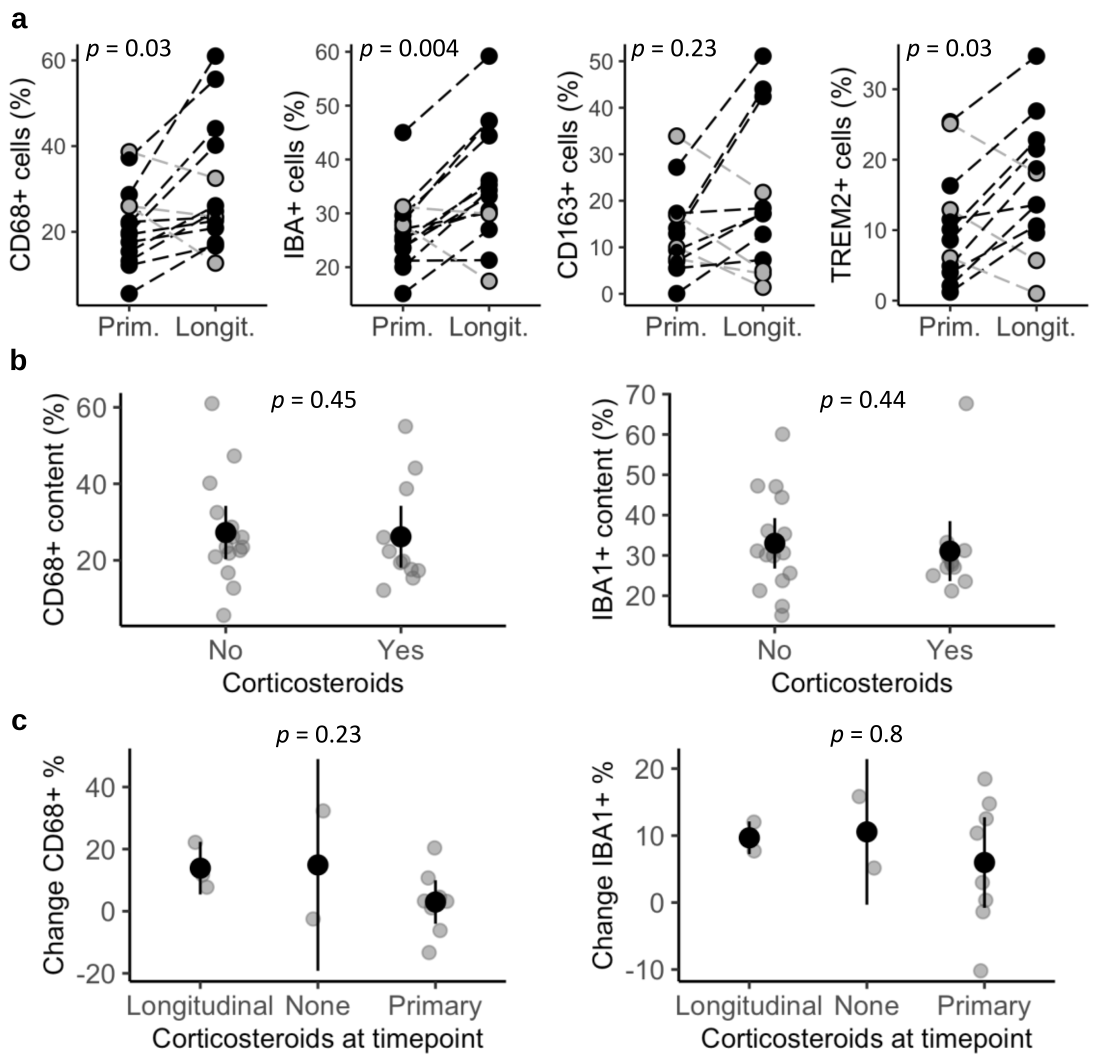

Supplementary Figure S3. CD68/IBA1 versus therapy with after exclusion of the single NF1 altered patient (**a**). Receipt of corticosteroids prior to surgery does not correlate with CD68+/IBA1+ content at the individual sample level (**b**) When patients were grouped according to timing of steroids, there was no effect on the size of CD68+/IBA1+ changes (**c**).

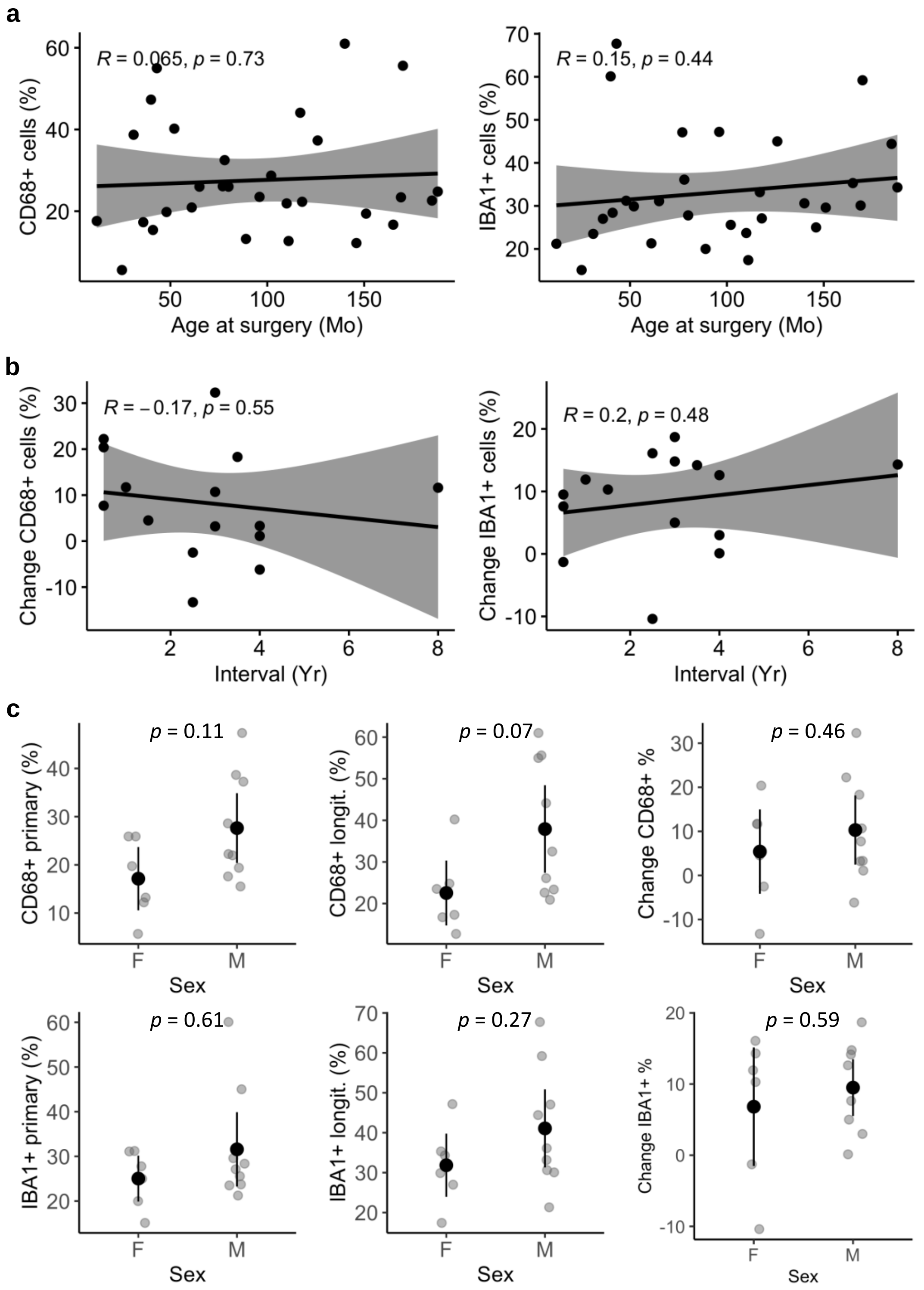

Supplementary Figure S4. CD68+/IBA1+ changes do not correlate with age at surgery (**a**) or interval between operations (**b**). Male patients trend towards higher CD68+ populations at both time points, but sex does not impact the size of CD68+/IBA1+ increases over time (**c**).

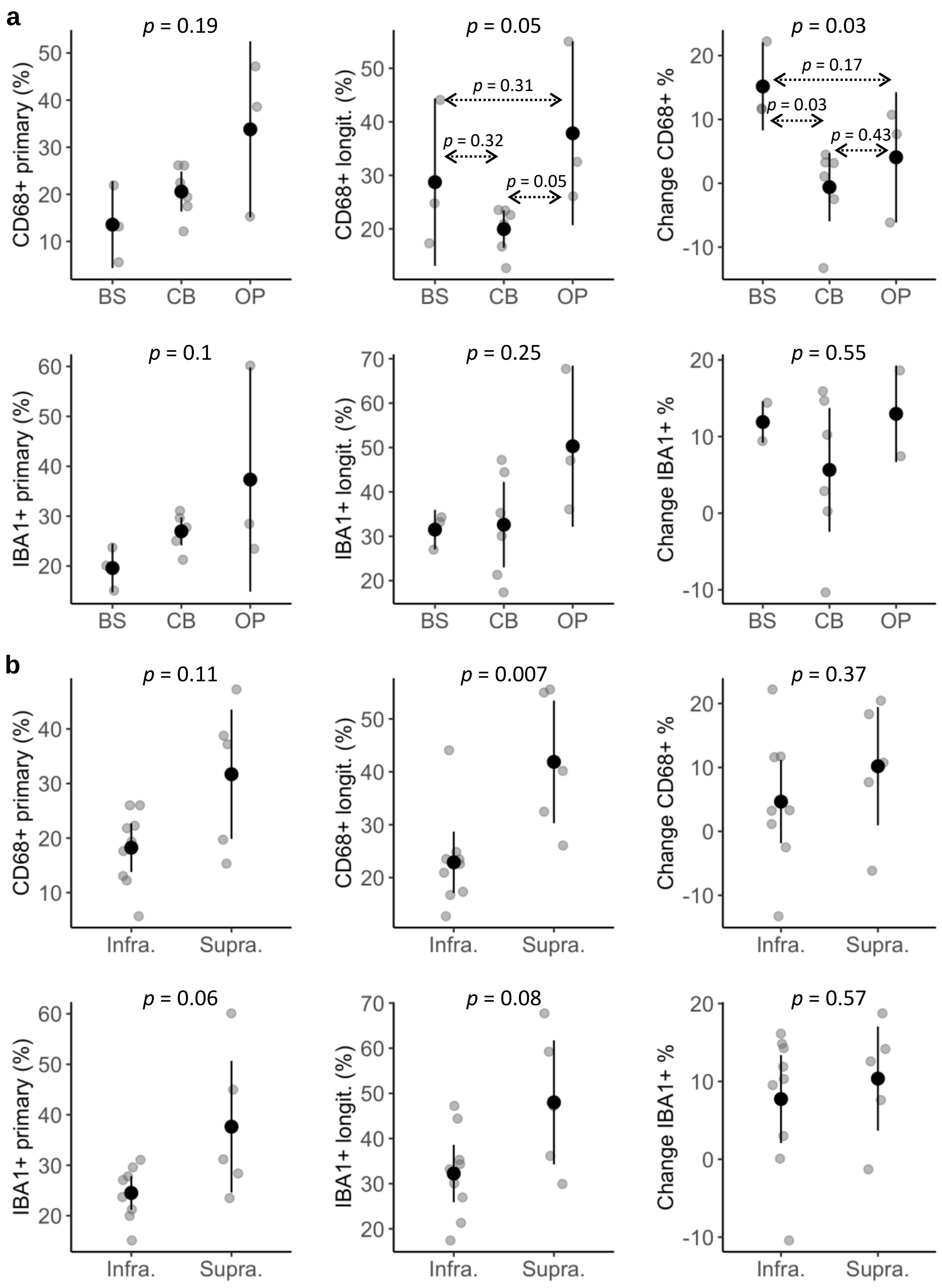

Supplementary Figure S5. Optic pathway tumours possess larger CD68+ populations than cerebellar, but not brain stem tumours at the longitudinal time point, while brain stem tumours show a trend for larger CD68+ increases over time than cerebellar but not optic pathway tumours (**a**). When divided into infra- and supratentorial regions, supratentorial tumours possess larger CD68+ populations at the longitudinal time point but here is no effect on the size of the CD68+/IBA1+ increase over time (**b**). BS = brain stem; CB = cerebellum; OP = optic pathway
